# Crystal Structure of Escherichia coli originated MCR-1, a phosphoethanolamine transferase for Colistin Resistance

**DOI:** 10.1038/srep38793

**Published:** 2016-12-13

**Authors:** Menglong Hu, Jiubiao Guo, Qipeng Cheng, Zhiqiang Yang, Edward Wai Chi Chan, Sheng Chen, Quan Hao

**Affiliations:** 1 School of Biomedical Sciences, University of Hong Kong, Laboratory Block, 21 Sassoon Road, Pokfulam, Hong Kong, China; 2 Shenzhen Key lab for Food Biological Safety Control, Food Safety and Technology Research Center, Hong Kong PolyU Shen Zhen Research Institute, Shenzhen, P. R. China; 3 State Key Lab of Chirosciences, Department of Applied Biology and Chemical Technology, The Hong Kong Polytechnic University, Hung Hom, Kowloon, Hong Kong

## Abstract

MCR-1 is a phosphoethanolamine (pEtN) transferase that modifies the pEtN moiety of lipid A, conferring resistance to colistin, which is an antibiotic belonging to the class of polypeptide antibiotics known as polymyxins and is the last-line antibiotic used to treat multidrug resistant bacterial infections. Here we determined the crystal structure of the catalytic domain of MCR-1 (MCR-1-ED), which is originated in *Escherichia coli (E. coli*). MCR-1-ED was found to comprise several classical β-α-β-α motifs that constitute a “sandwich” conformation. Two interlaced molecules with different phosphorylation status of the residue T285 could give rise to two functional statuses of MCR-1 depending on the physiological conditions. MCR-1, like other known pEtN transferases, possesses an enzymatic site equipped with zinc binding residues. Interestingly, two zinc ions were found to mediate intermolecular interactions between MCR-1-ED molecules in one asymmetric unit and hence concatenation of MCR-1, allowing the protein to be oligomer. Findings of this work shall provide important insight into development of effective and clinically useful inhibitors of MCR-1 or structurally similar enzymes.

The effectiveness of antibiotics to combat bacterial infections has diminished rapidly in the past decade due to incessant emergence of bacterial strains that exhibit novel and transmissible resistance mechanisms, such as carbapenem-resistant Enterobacteriaceae (CRE) strains which commonly cause untreatable and hard-to-treat infections among hospitalized patients. CRE is now considered an urgent public health threat according to a report by the Center for Diseases Control and Prevention (CDC) in 2013^1^. In the United States alone, more than 9,000 healthcare-associated infections are caused by CRE each year, and 50% of the patients who suffer from bloodstream infections are lethal^1^.

Polymyxin is currently considered a last-resort antibiotic which can be used to treat clinical CRE infections due to its high efficacy and low resistance rate among CRE. Bacterial resistance to polymyxin was thought to be low, and mainly attributed to chromosomal mutations in genes encoding specific two component regulatory systems (eg, PmrAB, PhoPQ, and its negative regulator MgrB in the case of *K. pneumoniae*), which lead to modification of lipid A or total loss of the lipopolysaccharide in the outer membrane^2^,^3^. Recently, a new plasmid-encoded colistin resistance mechanism, mediated by the MCR-1 protein (a phosphoethanolamine (pEtN) transferase that modifies the pEtN moiety of lipid A), has been discovered^4^. The *mcr-1* gene was found to be located in a plasmid which can self-transmit between animal and human isolates. Since its discovery in November 2015, the *mcr-1* gene has been reported in a wide range of bacterial species worldwide, suggesting that this resistance element is highly transmissible, posing a huge challenge to the use of polymyxin as a reserved drug for treatment of infections caused by CRE^5^. Most importantly, *mcr-1* positive *Enterobacteriaceae* strains have been detected in the gastrointestinal (GI) tract of human, including infants who have never been subjected to prolonged exposure to antibiotics. This phenomenon is suggestive of stable colonization of *mcr-1*-positive *Enterobacteriaceae* in the human GI tract even without antibiotic selection pressure^6^. Considering the transmissible nature of the *mcr-1* gene, the increasing prevalence of *mcr-1*-bearing organisms in the human GI tract is expected to further enhance the dissemination of this resistance gene among a wide range of bacterial species. Upon approval of clinical use of colistin in China and other regions of the world in the near future, we envisage that *mcr-1* will continue to be disseminated extensively in the hospital environment. The use of colistin to treat CRE infections may therefore result in rapid selection of organisms that exhibit resistance to both carbapenems and colistin. Development of effective inhibitors for MCR-1 may be the only effective strategy to prolong the use of colistin as the last-line antibiotic to treat life-threatening bacterial infections. The prerequisite for development of MCR-1 inhibitor is to depict the structure of this protein. We report herein, for the first time, the crystal structure of MCR-1 in order to provide insight into both the structure/function relationship of this novel enzyme and facilitate development of countermeasures to reverse the colistin resistance phenotypes in major bacterial pathogens.

## Results and Discussion

### Overall structure of MCR-1 extracellular domain

**Figure 1:**
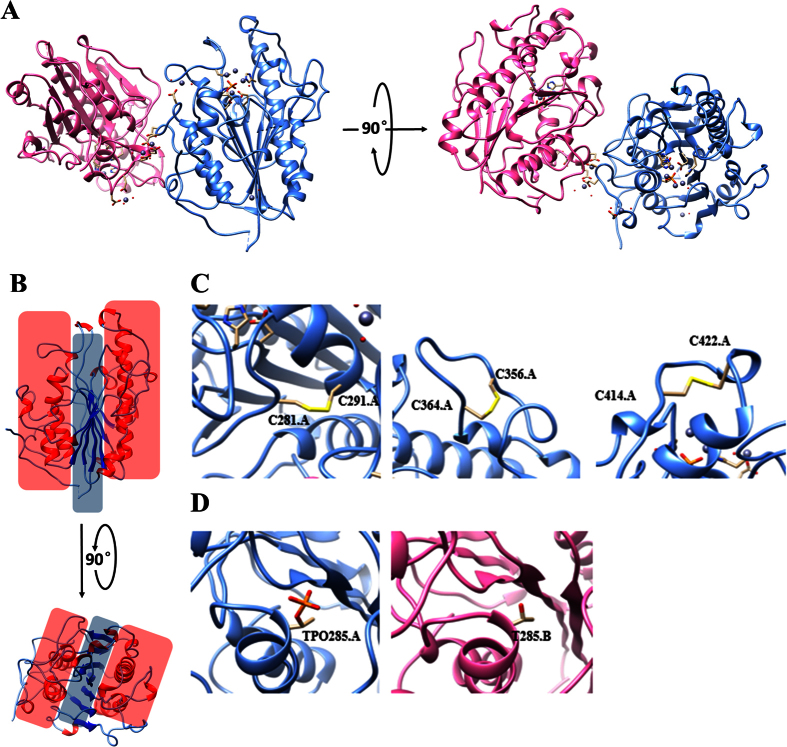
Overall structure of MCR-1-ED. (**A**) Front and top view of the crystal structure of MCR-1-ED. Two molecules in one asymmetric unit are labeled with cornflower blue and hot pink for chain A and B respectively. (**B**) Front and top view of the “sandwich” conformation of MCR-1-ED covered by red (α-helix layer) and blue (β-sheet layer) shadows. Secondary structures of α-helixes and β-stands are colored in red and blue respectively. (**C**) Three pairs of disulfide bonds of MCR-1-ED labeled with yellow on chain A. The other three are located at the same positions on chain B. (**D**) Threonine 285 of MCR-1-ED with and without phosphorylation.

MCR-1 is a potential pEtN transferase that exhibits ~40% sequence identity with LptA, which contains three domains (intracellular, transmembrane and extracellular), with the extracellular domain being the active transferase^4^. In this study, we aimed at determining the structure of the extracellular domain of MCR-1, namely MCR-1-ED, which comprises residues 200~540 based on the sequence alignment between MCR-1 (full length) and LptA (PDB code: 4KAY) (Supplementary Fig. 3). Upon protein purification and crystal optimization, we collected a diffraction data set at the Shanghai Synchrotron Radiation Facility and then used the crystal structure of LptA as the search model to solve the phase problem by molecular replacement^7^. After model rebuilding and refinement^8^,^9^ we finally determined the three dimensional structure of MCR-1-ED, which spanned residues T200 through I540, at 2.33 Å (Fig. 1A). Similar to LptA^10^, MCR-1-ED was found to comprise several classical β-α-β-α motifs which constitute a “sandwich” conformation, with one internal β-sheet layer and two ambilateral α-helix layers (Fig. 1B). The seven central β-strands, comprising six parallel and one reversed strands, were shown to be clamped by eight main α-helixes. Compared to the stable core of the “sandwich”, the interlinking loops of β-α-β-α motifs were much more flexible, despite the fact that three of them were anchored by three pairs of disulfide bonds (C^281^-C^291^, C^356^-C^364^ and C^414^-C^422^) (Fig. 1C). The stabilization effects conferred by the disulfide bonds could be disrupted by reductants such as β-mercaptoethanol or DTT, leading to inferior crystal diffraction with low resolution (data not shown). Each asymmetric unit contains two MCR-1-ED molecules. The two interlaced molecules are almost identical in their overall shape except at the potential catalytic sites T^285^ is phosphorylated in chain A but not in chain B (Fig. 1D) shown in omit density map as well (Supplementary Fig. 4), the structural details of which will be elaborated below. Occupancy of the phosphorylated T^285^ residue in chain A is 1.00 with low B-factor, whereas there is no appropriate electron density for fitting the phosphate group in T^285^ of chain B, inferring the existence of two different states of MCR-1 under physiological environment. In comparison, the structure of LptA was known to exist in the form of dimer, in which both nucleophilc T^280^ residues were found to be phosphorylated. However there were two states of the phosphate, one covalently bound to the side chain of threonine, whereas the other one existed in a free form near the threonine residue^10^. Undoubtedly, phosphorylation of the residue threonine in both LptA and MCR-1 played a pivotal role in substrate modification. Detection of different states of T^285^ in MCR-1 may reflect the existence of multiple reaction states during catalysis. Further investigation is required to elucidate the differential functional roles of the different reaction states of T^285^.

During the process of model building and refinement, we found that eleven sites in one asymmetric unit were filled with large globular densities, and that these undefined high density sites, located in close proximity to the histidines, aspartic acids and glutamic acids residues, resulted in high Rfree/Rwork ratio if they were not fitted with atoms. In view of the metal binding capability of LptA, we speculated that observation of these high density sites reflected the existence of some kinds of metal ions, since they were also shown in the phased anomalous difference map (Supplementary Fig. 1). Furthermore, zinc ions were needed to grow the MCR-1-ED crystal. To investigate the possibility that these sites were filled with zinc ions, we placed eleven zinc ions into the high density sites and found that they further lowered the Rfree/Rwork values after refinement. Moreover, we used a florescence scan at SSRF to detect zinc ions in crystal directly, and the result showed that MCR-1-ED contained zinc ions (Supplementary Fig. 5). To assess whether zinc ions have been artificially introduced during the process of MCR-1-ED protein crystal growth, we determined the metal content of the purified MCR-1-ED in the buffer-free of zinc ion using inductively coupled plasma optical emission spectroscopy (ICP-OES). The metal coordinated in the MCR-1-ED was confirmed to be zinc (Supplementary Fig. 2). The molar ratio between MCR-1-ED and zinc ion was approximately 3:1, which was different from that (2:11) in the crystal structure. The inconsistency between the MCR-1: zinc ion ratio in the MCR-1 crystal and the purified protein could be due to the following reasons. First, over-expression of MCR-1-ED in *E. coli* may result in insufficient supply of zinc ions derived from cells for coordination of all MCR-1 molecules. Second, the purification process using NTA column, in which an excess amount of imidazole was used, may cause depletion of some Zn ions in the purified MCR-1. Third, the buffer in which protein crystals grew contained overdose of zinc ions necessary for forming protein crystals. Due to the above reasons, the exact number of zinc ions in MCR-1 could not be measured, although there is sufficient evidence for us to conclude that MCR-1 is a zinc binding protein.

### MCR-1 possesses a potential active site for phosphoethanolamine transferase

**Figure 2:**
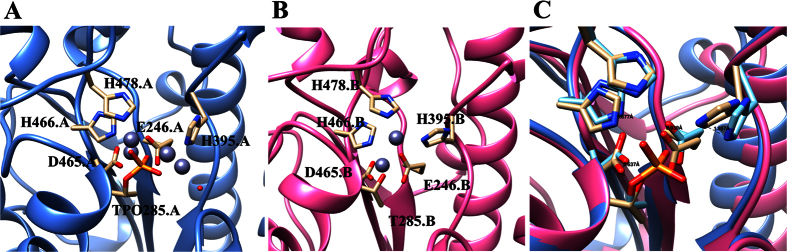
Enzymatic sites of MCR-1-ED. (**A** and **B**) Enzymatic centers of chain A with TPO285 and B with T285. Conserved residues important for Zinc ions (gray balls) binding are highlighted with O and N atoms colored in red and blue. The enzymatic centers of chain A with TPO285 could trap four Zinc ions (**A**), but chain B with T285 could only trap two. Water molecules are shown as small red balls. (**C**) Structural comparison between two types of enzymatic centers by superimposing. After restraining one α-helix where T285 resident, shifting distances of Zinc ion binding residues were measured by Chimera. Phosphorylation on T285 can create a patulous pocket and allows accommodation of more Zinc ions.

MCR-1 has been proven to be a phosphoethanolamine (pEtN) transferase which can transfer pEtN from the primary lipid phosphatidylethanolamine (PE) to lipid A^4^. Given the high degree of structural and sequence similarity between MCR-1 and other transferases, such as LptA and EptC^10^,^11^ we envisage that MCR-1 possesses a similar enzymatic site equipped with zinc binding and phosphorylated residues. In LptA, the metal (zinc) is tetrahedrally coordinated by H^453^, D^452^, E^240^ and T^280^, in which the T^280^ residue is phosphorylated^10^; in EptC, zinc ion is also coordinated tetrahedrally by D^427^, H^428^, E^227^ and phosphor-T^266^ 11. By sequence alignment and structural comparison (data not shown), the zinc ions in the MCR-1 were found to be hexagonally coordinated by E^246^, H^395^, D^465^, H^466^, and H^478^ and phosphorylated T^285^. The region harboring these residues was likely to constitute the active site of MCR-1 due to the presence of both a metal ion and the phosphorylated-T^285^ residue (Fig. 2A and B). Such residues are highly conserved in known phosphoglycerol transferases, such as LtaS in *Staphylococcus aureus*, although the metal ion harbored by LtaS is manganese^12^. In *E. coli*, pEtN transferase catalyzes the transfer of pEtN from primary lipid phosphatidylethanolamine (PE) to lipid A. LptA, a MCR-1 homologue, is a pEtN transferase which can catalyze the process of pEtN modification on lipid A in *Neisseria gonorrhoeae*. It has a putative enzymatic site equipped with zinc binding and phosphorylated residues. Protein sequence alignment between MCR-1 and LptA indicates that highly conserved residues are clustered around the zinc binding pockets including the five principal zinc ion binding sites (E^246^, H^395^, D^465^, H^466^ and H^478^) and one phosphorylation site (T^285^) in MCR-1. In the active site of one asymmetric unit of MCR-1-EDs, there are four and two zinc ions trapped by chain A and chain B respectively (Fig. 2A and B), suggesting that MCR-1 exhibits the capability to attract zinc ions at different levels. Consistent with the previous tests, T^285^ of chain A was phosphorylated but chain B was not. The negatively charged phosphate group was found to contribute to the coordination with two zinc ions. After superimposing chains A and B (Fig. 2C), we found that insertion of the phosphate moiety in T^285^ of chain A led to a shift of zinc ion binding residues with a displacement distance around 0.20–1.96 Å^13^. The structurally augmented enzymatic site then allowed more zinc ions to enter. Successful capture of both native and phosphorylated MCR-1-ED structures suggested that the MCR-1 protein, which contained a phosphorylated T^285^ residue, was in an intermediate state of pEtN to lipid A transfer. In addition, the relatively large number of metal ions captured by MCR-1, as compared with LptA, EptC or LtaS, may be due to the presence of more coordinative residues (six) in the active site of MCR-1.

To further confirm our structural prediction on the active site of MCR-1 and assess the functional requirement of these conserved residues, mutational analysis of these residues was performed. The results confirmed that the key roles of T^246^, D^465^, H^466^ and H^478^ in MCR-1 activity were mediated through coordination of active site zinc ion, as mutations that resulted in the T^246^A, D^465^A, H^466^A and H^478^A amino acid changes abolished the activity of MCR-1 and lowered the colistin MIC of *E. coli* to the same level as the control (BL21) (Table 1). It should be noted that although residue H^395^ could interact with the zinc ion located on the surface of the active site of MCR-1, it did not appear to contribute to the activity of MCR-1, suggesting either that this residue might not play a key role in Zn^2+^ ion coordination, or that the zinc ion located on the surface of the active site may not be critical for maintaining the activity of this protein. Other active site residues such as N^329^ and S^330^ were also not found to contribute significantly to the activity of MCR-1. The role of T^285^ was also tested through mutational analysis. The T^285^A mutation was found to cause reversal of the colistin resistance phenotype.

### Zinc ion induced MCR-1-ED oligomerization

**Figure 3:**
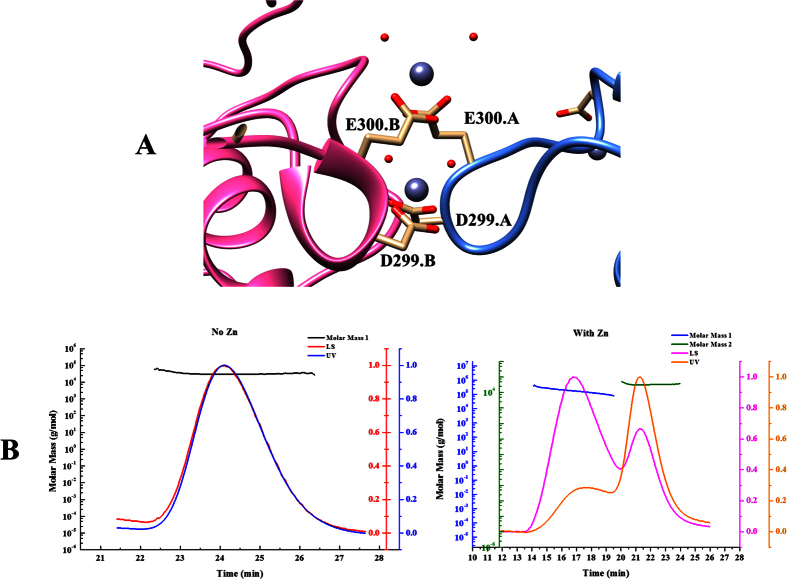
Homophilic interaction of MCR-1-EDs. (**A**) Interface of two MCR-1-EDs are mediated by two Zinc ions in one asymmetric unit. Residues that cooperate with Zinc ions are named on both chains. (**B**) Oligomerization of MCR-1-ED induced by Zinc ions. The left and right panels depict the status of MCR-1 in zinc-free buffer and buffer containing 1 mM zinc ion, respectively.

Besides the presence of six zinc ions in the potential enzymatic sites and three on the surface of MCR-1-ED, two zinc ions that interact with D^299^ and E^300^ of both chain A and B in one asymmetric unit were found to be located at the interface (Fig. 3A). We predicted that zinc ions played a role in mediating intermolecular interactions among MCR-1-ED molecules. To test whether zinc ions exhibit a catalytic effect on concatenation of MCR-1-ED molecules in solution, we utilized the Static Light Scattering (SLS) technique to assess the polymerization states of the purified protein in the presence and absence of zinc ions. Protein of high purity in zinc-free solution was found to exhibit one sharp symmetric peak in the UV curve upon gel-filtration. With horizontal result fitting, molecular weight (Mw) analysis revealed the presence of a ~35 kDa protein, which was equivalent to the Mw of the MCR-1-ED monomer (Fig. 3B, left panel). However, after incubation in zinc solution (1 mM), MCR-1-ED exhibited two peaks in the UV curve with one settling at the site as the monomer while the other emerged at an earlier position (Fig. 3B, right panel). In a gel-filtration column, molecules with large Mw come out before the ones with small Mw. Peak1 therefore represented a protein component with higher Mw than the MCR-1-ED monomer due to the addition of zinc ions. Both Peak1 and Peak2 had horizontal result fitting, and Mw calculation indicated that Peak1 and 2 corresponded to proteins with Mw of 197 kDa and 37 kDa, representing MCR-1-ED oligomer and monomer respectively. We speculate that MCR-1 may function as an oligomer, which depends on zinc ions. To further confirm whether the oligomerization is contributed to the function of MCR-1 *in vivo*, mutational analysis was performed on these residues with the results showing that mutants, D^299^A and E^300^A, were still functionally active suggesting that oligomerization of MCR-1 may not contribute to its activity *in vivo* or single mutation was not enough to disrupt the oligomerization.

## Conclusion

We report for the first time the crystal structure of the catalytic moiety of the newly identified phosphoethanolamine (pEtN) transferase, MCR-1, which can confer bacterial resistance to colistin via modifying lipid A. Like its homologues in the YhjW/YjdB/YijP superfamily, such as LptA of *N. meningitis* and EptC of the Gram-negative pathogen *C. jejuni*, MCR-1 was found to exhibit an α/β/α-sandwiched structure and coordinate divalent zinc ions in the active site via phosphorylation of the conserved residue threonine. Although more zinc ions in MCR-1 are coordinated when compared with LptA and EptC, only four residues, namely D^465^, H^466^, T^285^ and H^478^ in the active site of MCR-1, were found to play a role in catalysis. The overall sequence and structure similarity among MCR-1, LptA and EptC suggest that they share similar mechanistic features in lipid A modification, but the difference in phosphorylation states of the T^285^ and the number of coordinated zinc ions in the active site involved in induction of oligomerization of MCR-1 depicted a possible novel feature of action for MCR-1. Knowledge regarding the highly conserved and functionally important residues in the active sites of MCR-1 and its homologues shall provide important insight into development of effective inhibitors of MCR-1 or structurally similar enzymes in the future.

## Materials and Methods

### Vector cloning and mutagenesis

As predicted based on the secondary-structure predictions in the UniProt (access code: A0A0R6L508), the first N terminal 178 residues of the MCR protein were found to contain several transmembrane domains. In order to facilitate the expression and purification of the protein, the gene that encode MCR-1 (200–541aa) was amplified by primers F-MCR200 (5′-GATCGAGCTCTCGGTGGGTAAGCTTGCCAG-3′) and R-MCR (5′-TCAGGGATCCTCAGCGGATGAATGCGGTGCG-3′) by using the plasmid pHNSHP45 (GenBank: KP347127.1) as template. The amplified gene was sub-cloned into pET-15b vector via the SacI/BamHI restriction sites. The identity of the construct was verified by DNA sequencing (BGI, China). Point mutations were introduced into the *mcr-1* gene by using the QuickChange (Stratagene) commercial kit following the manufacturer’s instructions and confirmed by sequencing.

### Protein expression, purification and crystallization

The expression vector pET-15b-MCR-1200–541 was transfected into the *E. coli* BL21 (DE3) RIL strain. Transfected cells were cultured in DYT media at 37 °C. Protein expression was induced by adding 0.3 mM IPTG until OD600 reached 0.8 at 16 °C overnight. Cell culture was centrifuged at 4000 rpm at 4 °C for 30 mins. Cell pellet was collected, re-suspended by Lysis buffer (20 mM Tris-HCl pH = 8.0, 500 mM NaCl and 10 mM imidazole), and lysed by sonicator. Lysed sample was centrifuged at 17,000 rpm at 4 °C for 30 mins. Supernatant was loaded onto Ni-column (GE) equilibrated by lysis buffer. After washing the column by Wash buffer (20 mM Tris-HCl pH = 8.0, 500 mM NaCl and 20 mM imidazole), protein was eluted by Elution buffer (20 mM Tris-HCl pH = 8.0, 500 mM NaCl and 500 mM imidazole). Eluted sample was dialysed in Dialysis buffer (20 mM Tris-HCl pH = 8.0 and 50 mM NaCl) at 4 °C overnight and then loaded onto Q-HP column (GE) equilibrated with Dialysis buffer (Buffer A). The protein sample was eluted in gradient manner with Buffer B (20 mM Tris-HCl pH = 8.0 and 1000 mM NaCl). Target sample fractions were collected, concentrated and loaded onto Superdex 75 (GE) equilibrated with buffer ((20 mM Tris-HCl pH = 8.0 and 200 mM NaCl) for further purification followed by SDS-PAGE identification. Target protein with high purity was pooled and concentrated to 70 mg/ml which was used for crystal screening and optimization. Crystal screening through vapor diffusion method was based on commercial kit from Hampton research. Sitting drop was performed by mixing equal volume of protein and reservoir. Condition hit (18% PEG8000, 0.1 M Sodium cacodylatetrihydrate pH = 6.5 and 0.2 M Zinc acetate dihydrate) was obtained after three days incubation at 18 degrees. Large crystals were grown in conditions around primary hit (10–20% PEG8000, 0.1 M Sodium cacodylatetrihydrate pH 6.2 and 0.2 M Zinc acetate dihydrate), which was seeded with diluted crushed crystals.

### Data collection, processing and structure refinement

Crystals were trapped in loops with cryoprotection agents, flash vitrified in liquid nitrogen, and taken to Shanghai Synchrotron Radiation Facility (SSRF) for diffraction data collection at 100 K on beamline BL17U. Data were processed with HKL2000^14^, followed by molecular replacement through Phaser in the CCP4 suit^7^. The MCR-1 homologue LptA (4KAY) was used as a search model. Model rebuilding was performed by Autobuild in the Phenix program suite^9^. Cycled manual model building in COOT was refined by the Phenix_Refine program^8^. The crystallographic statistics are listed in Supplementary Table1. A florescence scan was performed based on crystals in the cryoprotectant without zinc ions.

### Static Light Scattering

The SEC-MALS model was used for Static Light Scattering analysis. Size exclusion chromatography (SEC) was first used for isolating proteins with different polymerization status and set before Multi-Angle Light Scattering analysis (MALS). Gel filtration column was equilibrated with Zinc-free buffer (20 mM Tris, pH 8.0, and 200 mM NaCl) and Zinc buffer 20 mM Tris, pH 8.0, and 200 mM NaCl, 1 mM Zinc acetate) respectively. Purified MCR-1-ED was incubated in these two buffers. After loading the sample onto the column, UV and correlated scattering signal were collected by FPLC (GE) and DAWN HELEOS (Wyatt Technology Corporation). Data analysis and Mw calculation were done by the ASTRA software (Wyatt Technology Corporation).

### Colistin susceptibility test

The full-length *mcr-1* gene was amplified by following the above protocol except that the primers F-MCR (GATCGAGCTCATGATGCAGCATACTTCTGTG) and R-MCR (TCAGGGATCCTCAGCGGATGAATGCGGTGCG) were used. The amplification product was sub-cloned into a pET-15b vector and transferred into the *Escherichia coli* strain BL21. All the mutated derivatives of *mcr-1* were created by using QuickChange (Stratagene) commercial kit following the manufacturer’s instructions and confirmed by sequencing (BGI, China). According to the CLSI^15^, the colistin susceptibility of all the *mcr-1*-bearing strain and the corresponding mutants were performed by using micro-dilution method in the presence of 1 mM IPTG. *E. coli* strain ATCC 25922 was used as a quality control. The results were determined as MICs.

### Metal content determination

Analysis of the metal content in the MCR-1 protein was performed as previously reported^16^. Briefly, purified MCR-1 protein was dissolved in 20 mM Tris, pH 8.0, and 200 mM NaCl. Zinc and indium (internal control) were chosen as standard metals; >0.99 correlation coefficient standard calibration curves were obtained using a series of BDH metal standards. The blank buffer was 1% Nitric Acid with 5 ppm Indium. All the MCR-1 protein and standards were diluted accordingly with the blank buffer. The metal signals were measured by the inductively coupled plasma optical emission spectroscopy (ICP-OES) (Santa Clara, CA) with 3 replicates for each sample.

### Data Deposition

Coordinates and the structural factors of the MCR-1 crystal structure has been deposited to PDB under code 5GOV.

## Additional Information

**How to cite this article**: Hu, M. *et al*. Crystal Structure of Escherichia coli originated MCR-1, a phosphoethanolamine transferase for Colistin Resistance. *Sci. Rep.* **6**, 38793; doi: 10.1038/srep38793 (2016).

**Publisher's note:** Springer Nature remains neutral with regard to jurisdictional claims in published maps and institutional affiliations.

## Supplementary Material

Supplementary Information

## Figures and Tables

**Table 1:** The colistin MIC of *E. coli* strain BL21 carrying the wild type *mcr-1* gene and mutation-containing fragment which encode amino acids 1–541 of the MCR-1 protein.

*E. coli* BL21 Strains	Colistin MIC (μg/ml)
BL21	2
pET15b	2
pET15b-*mcr-1*FL*	8
pET15b-*mcr-1*FL (T246A)	2
pET15b-*mcr-1*FL (T285A)	2
pET15b-*mcr-1*FL(D465A)	2
pET15b-*mcr-1*FL(H466A)	2
pET15b-*mcr-1*FL(H478A)	2
pET15b-*mcr-1*FL(H395A)	8
pET15b-*mcr-1*FL(N329A)	8
pET15b-*mcr-1*FL(S330A)	8
pET15b-*mcr-1*FL(D299A)	8
pET15b-*mcr-1*FL(E300A)	8

^*^mcr-1FL represents MCR-1(1–541).
