# Supplementary material for: Crystal Structure of Escherichia coli originated MCR-1, a phosphoethanolamine transferase for Colistin Resistance
Source: Sci Rep. 2016 Dec 13;6:38793. doi: 10.1038/srep38793 (PMC5153839; doi:10.1038/srep38793)
Supplement: Supplementary Information [file srep38793-s1.pdf]

Supplementary materials

Crystal Structure of Escherichia coli originated MCR-1, a phosphoethanolamine transferase for Colistin Resistance

Menglong Hu[a#], Jiubiao Guo[b,c#], Qipeng Cheng[b,c], Zhiqiang Yang[b,c], Edward Wai Chi Chan[b,c], Sheng Chen[b,c*], Quan Hao[a*]

[a] School of Biomedical Sciences, University of Hong Kong , Laboratory Block, 21 Sassoon Road, Pokfulam, Hong Kong, China;

[b]Shenzhen Key lab for Food Biological Safety Control, Food Safety and Technology Research Center, Hong Kong PolyU Shen Zhen Research Institute, Shenzhen, P. R. China;

[c]State Key Lab of Chirosciences, Department of Applied Biology and Chemical Technology, The Hong Kong Polytechnic University, Hung Hom, Kowloon, Hong Kong;

# These authors contributed equally.

[*] To whom correspondence may be addressed: E-mail:qhao@hku.hk or sheng.chen@polyu.edu.hk.

Supplementary Table 1. Crystallographic Data and Refinement Statistics

**Data Collection**	
	MCR-1-ED
Cell dimensions<br>a, b, c (Å)<br>α, β, γ (°)	 54.4, 55.8, 219.4<br>90, 90, 90
Space group	$P2_12_12_1$
Wavelength (Å)	0.9790
Resolution (Å)[a]	50.00-2.33 (2.37- 2.33)
Unique reflections	29251 (1160)
Redundancy	11.5 (9.8)
<I /σI>	22.27 (2.84)
Rsym	0.104(0.582)
Completeness (%)	97.5 (77.6)
**Refinement**	
Rwork	0.186
Rfree	0.236
RMSD from ideal<br>Bond lengths (Å)<br>Bond angles (°)	 0.009<br>1.184
Average B-factors ($Å^2$)	35.5
Ramachandran plot (%):<br>favored/allowed/outliers	 96.22/3.46/0.31
[a]Values in parentheses are from the highest-resolution shell.	

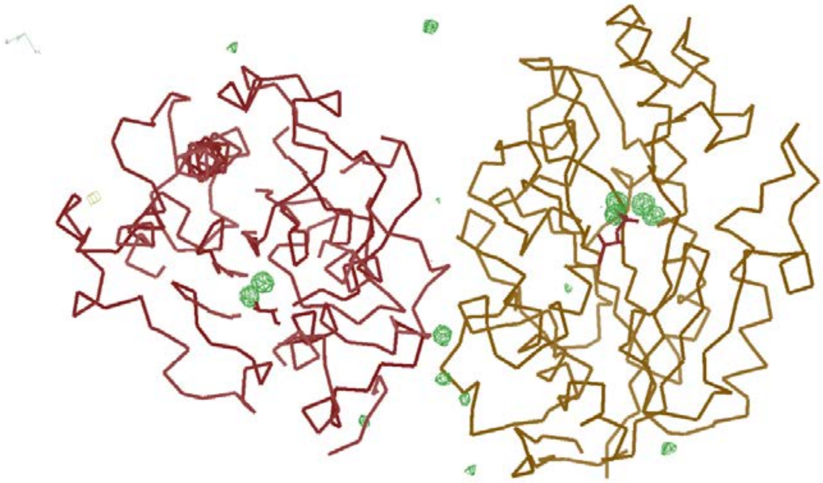

Supplementary Figure 1. Phased anomalous difference map (5.0 r.m.s.d.)

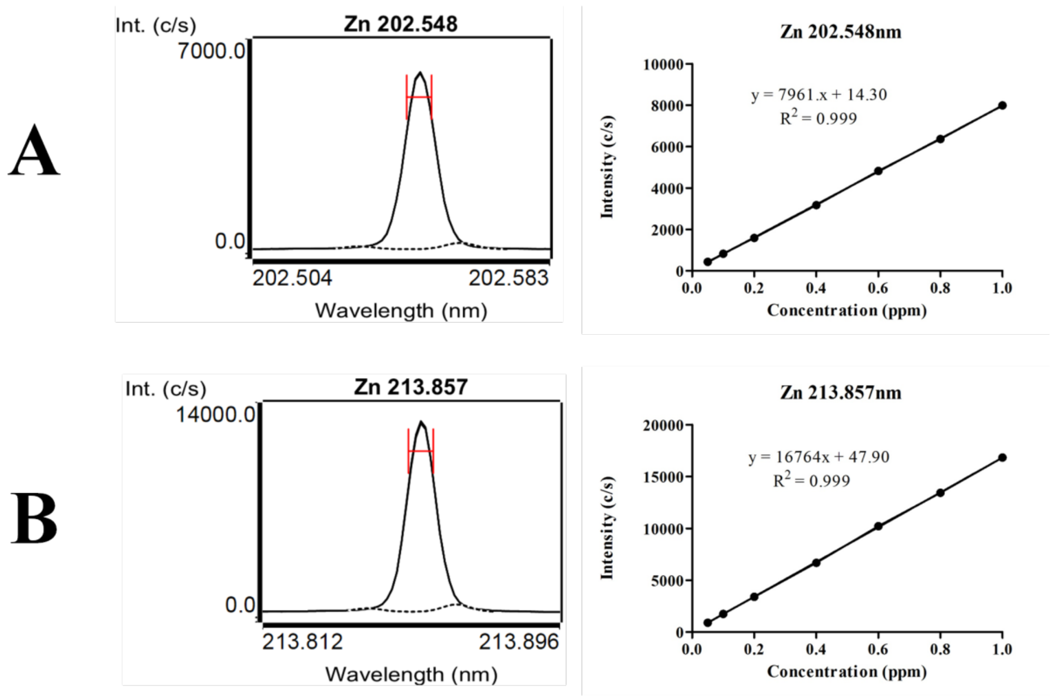

Supplementary Figure 2. Metal content determination in MCR-1 protein. The Zn content measurement at the wavelength of 202.548nm (A) and 213.857nm (B). The left panels show the Zn content in the MCR-1 protein sample, and the right panels depict the corresponding Zn standard curves compiled for determining the Zn concentration in the MCR-1 protein sample.

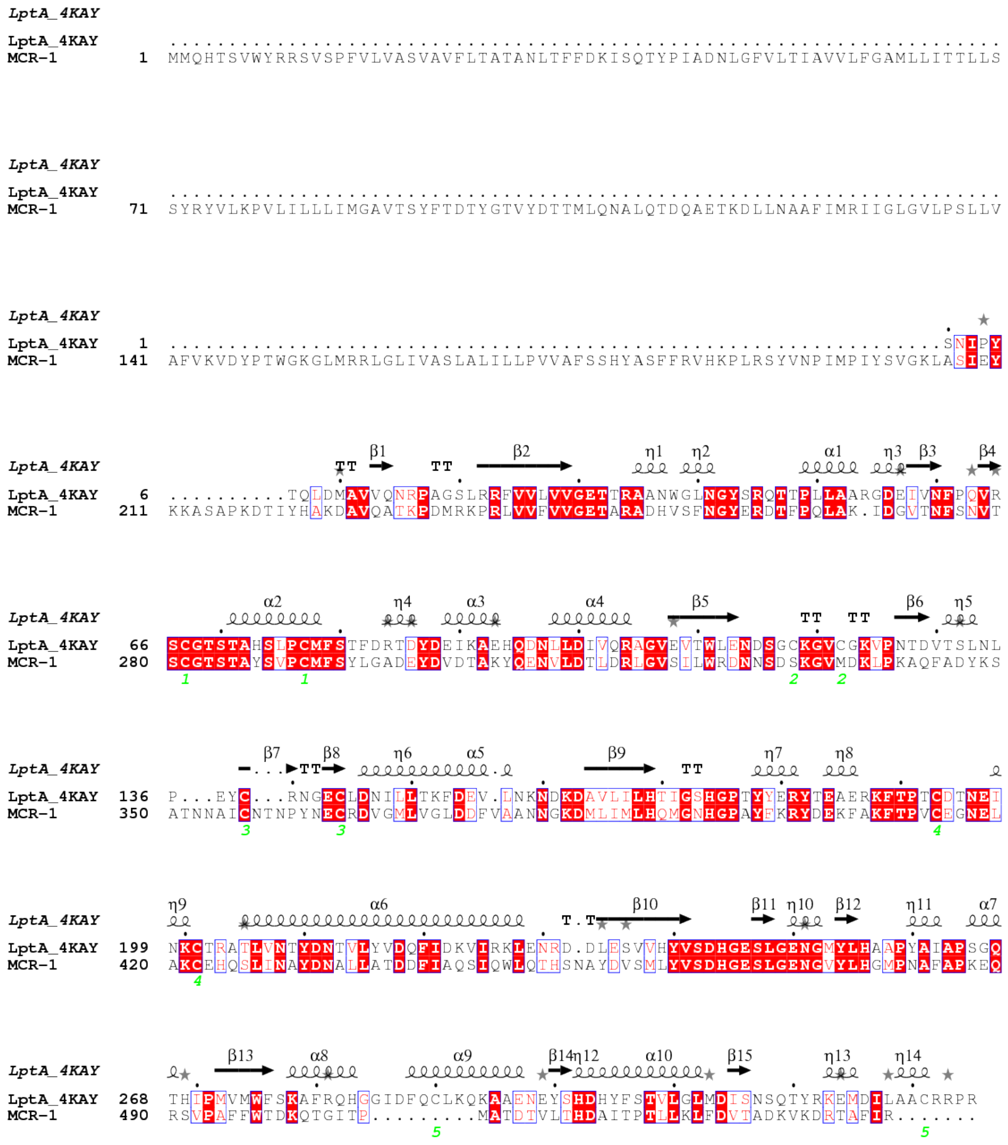

Supplementary Figure 3. Sequence alignment between MCR-1 (full length) and LptA (PDB code: 4KAY).

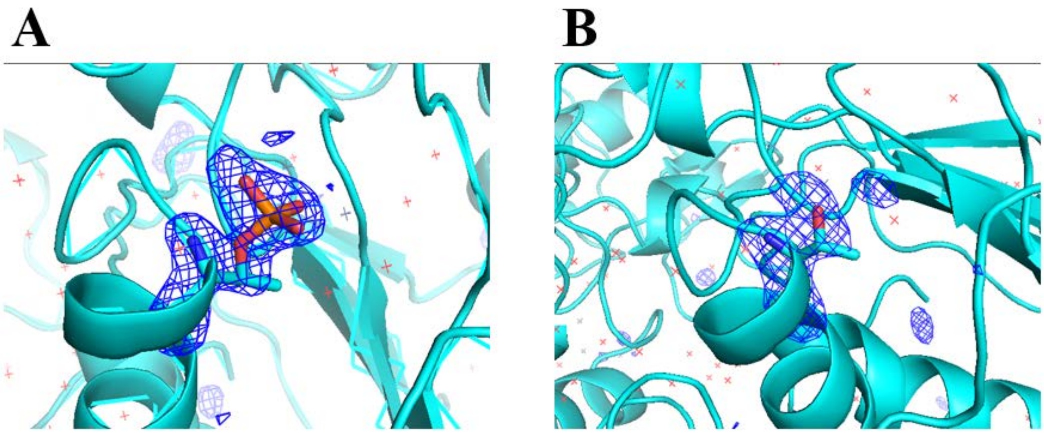

Supplementary Figure 4. The Omit-map density of the phosphorylation site at T285 of both chain A and B. (A) Phosphorylated T285 on chain A; (B) Non-phosphorylated T285 on chain B.

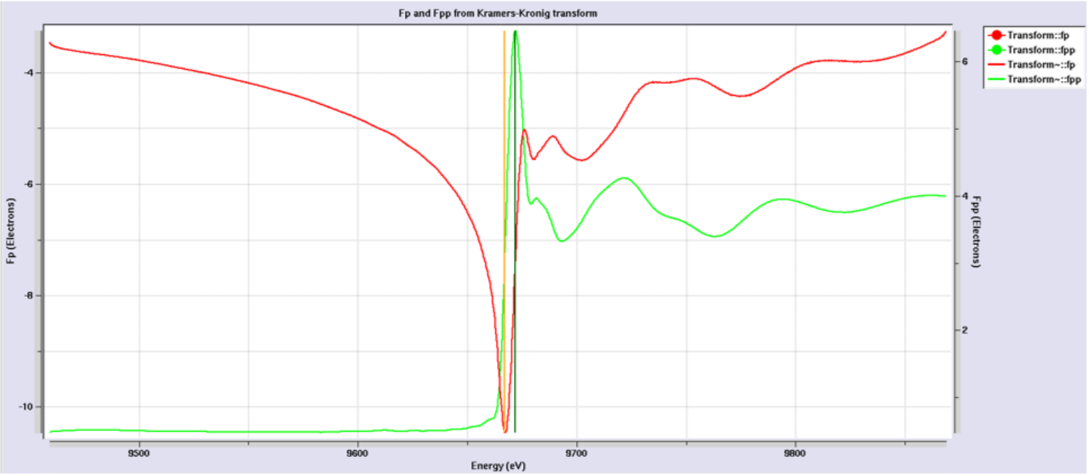

Supplementary Figure 5. Florescence scan of Zinc ions in crystals.
